# Effect of some growth substances application on growth and seasonal yield of acid lime trees (*Citrus aurantifolia* Swingle) in Delta

**DOI:** 10.1038/s41598-025-87431-8

**Published:** 2025-02-07

**Authors:** Waleed Fouad Abobatta, Mahmoud A. Khodier, Hassan S. H. Ismail

**Affiliations:** Horticulture Research Institute, Agriculture Research Center, Giza, 12112 Egypt

**Keywords:** Chitosan, Fruit quality, Hydrogen peroxide, Lemon trees, Yield, Ecology, Plant sciences, Plant physiology

## Abstract

This work aims to investigate the impact of chitosan and hydrogen peroxide on the growth and seasonal yield of lime trees cultivated in commercial orchards in Qalyubiyya Governorate, Egypt, during the 2021 & 2022 seasons. The treatments include foliar spraying of two concentrations of CHI (100 & 200 ppm) and H_2_O_2_ (2 & 4 cm/L), either individually or in combination, at two distinct times, one month after fruit set (1st week of September) and after two months. The hypothesis was that applying chitosan and hydrogen peroxide would improve tree growth, fruit quality, and total production. The obtained results indicated that the combinations of CHI with H_2_O_2_ improved tree growth, leaf mineral composition, seasonal yield, and fruit quality parameters. CHI at 200 ppm + H_2_O_2_ at 4 cm/L was the most efficient treatment and achieved the largest tree canopy. Treatment of 100 ppm CHI with 2 cm/L H_2_O_2_ showed superior yield in terms of tree yield (23.56 & 29.64 kg/tree), total production (8.15 & 9.16 t/ha) compared to other treatments. Likewise, combinations of CHI and H_2_O_2_ improved fruit characteristics. Findings of this study demonstrated that the foliar application of CHI with H_2_O_2_ could be a promising application to improve seasonal lemon yield and fruit quality in commercial orchards.

## Introduction

Citrus is a highly nutrient-responsive tree, hence, nutrition plays a major role in determining tree productivity. Growth substances could play an important role in improving tree growth and productivity. Acid Lime (*Citrus aurantifolia* Swingle), one of the Citrus fruits belonging to *Rutaceae* family, has gained more attention worldwide due to its nutritional values, particularly its higher content of vitamin C. It is well known that lime fruits are not only for fresh use but also can be made into some industrial materials and medicines. Thus, the content of chemical constituents can represent the internal quality of lime fruit. The lime tree is a subtropical fruit and can bloom and produce fruit frequently throughout the year. Trees grow quickly and need great amounts of nutrients. Enhancing tree growth and productivity is crucial for addressing rising demands for fruit worldwide. Numerous external and internal factors affect citrus growth and productivity. Therefore, the use of supplement fertilizers directly affects the productivity and quality of the crop. Growth stimulants have an efficient impact on improving the productivity of fruit trees^1^. In citriculture, plant growth stimulants have become a crucial factor in increasing productivity by regulating flowering and fruit sets. They are considered effective compounds for regulating vegetative growth and fruit development by controlling endogenous processes, modifying the response of external growth through adapting vegetative growth with tree crops and preserving fruit quality^2^. Chitosan (CHI), is a natural polymer produced from chitin that is inexpensive and safe^3^. Chitosan acts as a biostimulant and influences metabolic pathways and improving tree yield through various mechanisms. It promotes cell division and elongation, improving photosynthesis by increasing energy capture and regulating hormonal balances that promote flowering and fruit set, thus increasing tree yield and enhancing fruit quality^4^. Since the 1980s, chitosan has been used in agriculture, and experiments have shown positive results on plant growth in addition to controlling many diseases in different crops^5,6^. Hydrogen peroxide (H₂O₂) acts as a signalling molecule in many biological processes. It regulates pathways such as cell proliferation and flower differentiation and oxidizes particular cysteine residues in proteins. At low levels, H₂O₂ has a significant role in cell cycle regulation and immune responses, thus enhancing flowering and total yield^7^. Both hydrogen peroxide (H₂O₂) and chitosan (CHI) act as growth promoters that enhance nutrient uptake. Foliar application of both substances to leaves ensures quick absorption and increased efficiency. The individual use of CHI and H₂O₂ on the growth and productivity of lemon trees has been studied several times. It is well documented that the use of CHI and H_2_O_2_ to enhance plant growth and obtain greater yields for various plants, so far, CHI and H₂O₂ have been widely used on different crops, and their positive effect on crop growth and quality has been confirmed. Studies on Washington Navel oranges by^8,9^, on mango trees by^10^, and on peach trees by^11^, reported the beneficial effect of CHI application. The application of H_2_O_2_ improves the growth and productivity of numerous crops, on mango trees by^12^, on canola plants by^13^, and on wax apple by^14^. While most of the previous studies on the effects of CHI or H₂O₂ on fruit crops have been done, the information available to the authors indicates that the nutritional effects of a combination of both CHI and H₂O₂ on the growth and productivity of lime trees have not been studied before under Egyptian conditions. CHI and H_2_O_2_ treatments as growth substances are not commonly used in the experimental region, and farmers in the study region are less aware of their use and very few have been studied on other citrus varieties. Nevertheless, research on the systematic evaluation of its specific effect on lime fruits is very limited. This work aims to improve the productivity of the seasonal crop productivity of acid lime trees by foliar spraying of both CHI and H₂O₂ or their combinations at different concentrations on plant growth, yield, and fruit quality as a novel application that increases production and enhances the profitability of acid lime farmers in Egypt.

## Materials and methods

A field experiment was carried out during two seasons (2020/21 and 2021/22) on 7-year-old lime trees (*Citrus aurantifolia* Swingle) budded on Volkamer lime (*Citrus volkameriana*). Planted at 5 × 5 m apart (400 trees/ha) and grown under a drip irrigation system with two adjustable emitters/trees (8 L/ha) through two irrigation lines in clay soil in a private orchard in Qalyubiya governorate, Egypt. Twenty-seven fruitful lime trees were selected based on uniformity in their size, shape, and disease-free status, the same trees were used for the experiment in both seasons. The experiment was laid out in a randomized complete block design (RCBD) that which, includes 3 blocks (3 replicates) and every block (replicate) contain nine treatments randomly distributed. This experiment was conducted to investigate the effect of foliar sprays of two substances, chitosan (100 and 200 ppm/L) and hydrogen peroxide (2 and 4 cm/L), on tree growth, leaf mineral content, seasonal yield, and fruit quality of acid lime grown in the Delta region. Other management practices were applied based on guidelines from the Ministry of Agriculture and Land Reclamation, Egypt. During the experimental seasons, the treatments were as follows: the control was sprayed with water, each substance was used as a separate treatment, and their combinations were used twice, one month after fruit set (1st week of September) and two months later (1st week of November) in a commercial orchard under the same conditions.

Treatments were used, as follows:

T1: Control (spraying with tap water).

T2: 100 ppm CHI.

T3: 200 ppm CHI.

T4: 2 cm/L H_2_O_2_.

T5: 4 cm/L H_2_O_2_.

T6: 100 ppm CHI + 2 cm/L H_2_O_2_.

T7: 100 ppm CHI + 4 cm/L H_2_O_2_.

T8: 200 ppm CHI + 2 cm/L H_2_O_2_.

T9: 200 ppm CHI + 4 cm/L H_2_O_2_.

Tree canopy volume was determined at the end of September for each season according to the equation of^15^.

Canopy volume = 0.52 × tree height × (diameter^2^).

Leaf mineral contents: at the end of September of every season, samples of 25 leaves were randomly picked from the middle part of non-fruiting spring shoots from the outer canopy of each replicate of the same trees each season. Wet digestion of plant materials was done, and leaf mineral contents of N, P, K, Fe, and Zn were estimated according to^16^.

### Testing Index of yield and method as well as fruit properties

Seasonal yield parameters were tree yield, total yield (ton/ha), fruit weight (g), fruit size (mm), fruit density, juice weight (g), juice ratio, TSS, acidity ratio, and yield efficiency. The tree yield was estimated as Kg/tree during harvesting, and total yield (ton/ha) was calculated theoretically. A sample of 25 fruits per replicate was selected randomly to determine the fruit’s physical properties according to^17^ and chemical characteristics according to^18^. Yield efficiency as fruit weight (kg)/m3 of canopy was recorded annually according to the equation of^19^.

Soil samples were analyzed before starting the experiment to determine the physical and chemical properties of the soil according to^20^, and they are presented in Table (1).

**Table 1:** Some physical and chemical properties of the tested soil.

Particle size distribution			
Clay %		51.3	
Sand %		15.3	
Silt %		33.4	
Soil Texture		Clay	
Chemical analysis			
PH ( 1 : 2.5 )		7.50	
EC ( 1 : 5 ) mmohs / cm		0.71	
O.M. %		1.50	
Soluble cations meq/ L.		Soluble Anions meq/ L	
Ca^++^	3.0	CO_3_^−−^	0.0
Mg^++^	1.6	HCO_3_^−^	2.4
Na^+^	2.8	Cl^−^	1.7
K^+^	0.7	SO_4_^−−^	4.0

### Statistical analysis

A randomized complete block design (RCBD) was used. The obtained data were subjected to the analysis of variance by ANOVA according to^21^. A statistical analysis was performed by computer software called MSTAT-C^22^. Differences between means were compared using Duncan’s multiple-range test at probability level of 0.05, according to^23^.

## Results

**Fig. 1:**
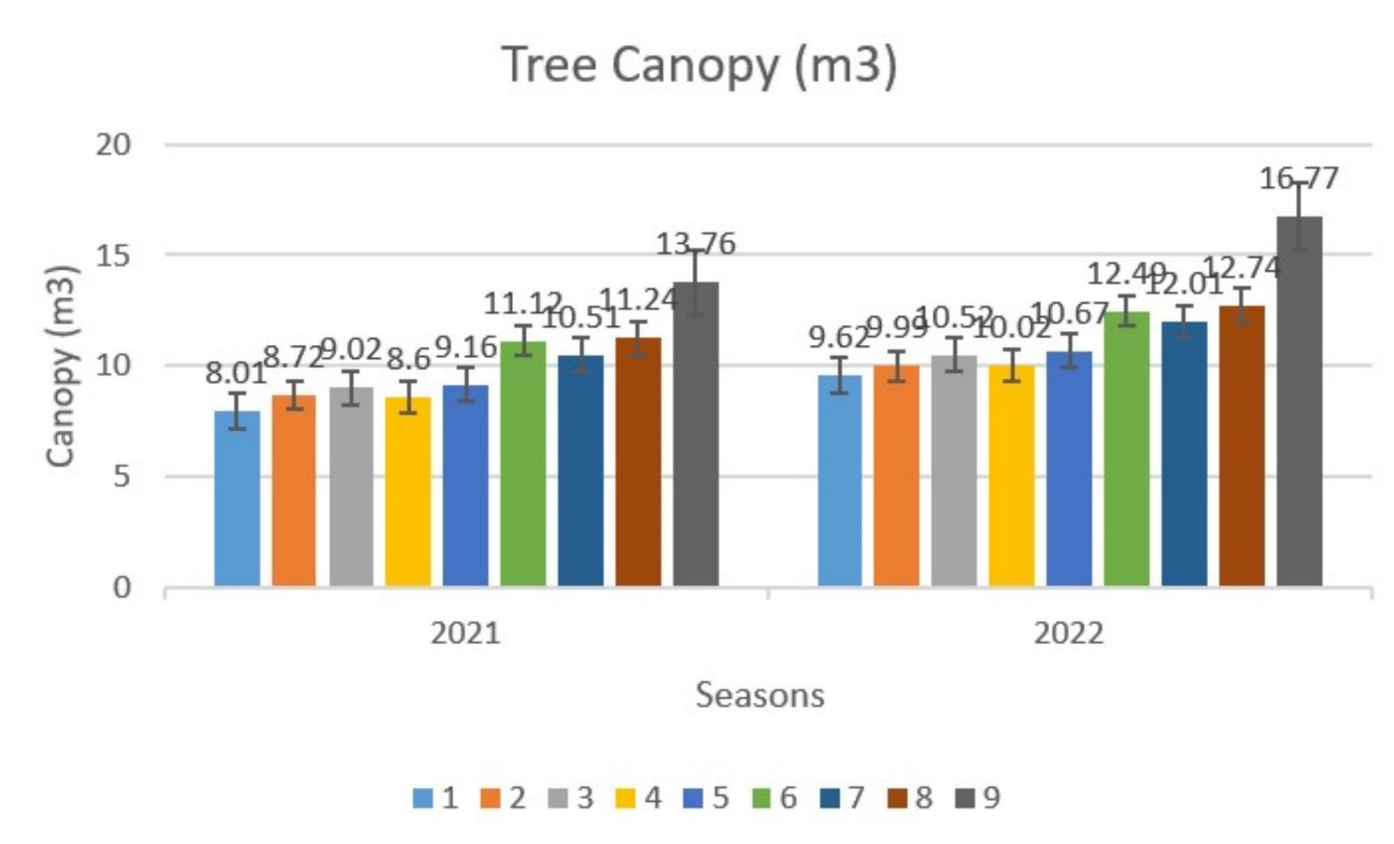
Effect of variance treatments on tree canopy of acid lime trees. * T1 (Control), T2 (100 ppm CHI), T3 (200 ppm CHI), T4 (2 cm/L H_2_O_2_), T5 (4 cm/L H_2_O_2_), T6 (T2 + T4), T7 (T2 + T5), T8 (T3 + T4), T9 (T3 + T5).

Data in (Fig. 1) indicated that single spraying of CHI and H_2_O_2_ concentrations had a significant effect on tree canopy during experimental seasons. Results showed that the combination of high concentrations of CHI with H_2_O_2_ significantly increased the tree canopy compared to control trees in the two successive seasons. The largest canopy size was recorded from trees subjected to T9 (13.76 & 16.77 m3), followed by T8 (11.24 & 12.74 m3), while, untreated trees (T1) recorded the lowest canopy size (8.01 & 9.62 m3). Furthermore, significant differences between treatments and control were detected in both seasons.

It is clear from Table (2) that exogenous application of CHI and H_2_O_2_ affected leaf chemical constituents, and both macro and micronutrients increased, which enhanced tree nutrient status but induced differences compared to untreated trees. Applications of combinations of CHI with H_2_O_2_ increased the accumulation of nutrients in the leaves compared to individual applications. The effect of treatments on the accumulation of elements varied without a similar trend during both seasons.

**Table 2:** Effect of variance treatments on leaf mineral contents of lime trees.

Treatment	*N* %		*P* %		K%		Fe mg/kg		Zn mg/kg		Mn mg/kg	
	2021	2022	2021	2022	2021	2022	2021	2022	2021	2022	2021	2022
T1	1.890 C	1.990 C	0.128 AB	0.129 AB	1.120 B	1.163 D	60.13E	60.96E	30.34E	30.61 F	31.92 H	31.29 F
T2	1.973 BC	1.993 C	0.122 B	0.125 B	1.183 AB	1.193 BCD	61.82CD	61.48E	30.50DE	30.93EF	33.97 F	33.80E
T3	2.017 ABC	1.990 C	0.127 AB	0.126 B	1.173 B	1.170 CD	61.32DE	61.86DE	30.83DE	31.10D-F	36.36E	35.00D
T4	1.990 ABC	2.017 C	0.126 AB	0.128 AB	1.200 AB	1.190 BCD	61.17DE	61.58E	30.60DE	31.45DE	32.73G	31.60 F
T5	2.070 AB	2.030 BC	0.129 AB	0.131 AB	1.160 B	1.183 BCD	62.90 C	63.18D	31.10D	31.67CD	38.50B	37.67B
T6	2.083 AB	2.107 AB	0.125 AB	0.130 AB	1.193 AB	1.207ABCD	66.40B	66.57B	34.54 A	35.72 A	37.80 C	36.37 C
T7	2.040 AB	2.047 ABC	0.133 A	0.136 A	1.187 AB	1.227 AB	63.15 C	64.9 C	33.77B	34.12 A	36.97D	35.30D
T8	2.087 AB	2.103 AB	0.131 AB	0.134 AB	1.180 AB	1.223 ABC	67.39B	64.70 C	33.50B	34.07B	39.70 A	38.33 A
T9	2.12 A	2.13 A	0.124 AB	0.126 B	1.230 A	1.250 A	68.97 A	69.33 A	32.02 C	32.17 C	38.13BC	37.23B

*Values in the same column followed by the same letter(s) do not significantly differ from each other according to Duncan’s multiple range test at 5% level.

* T1 (Control), T2 (100 ppm CHI), T3 (200 ppm CHI), T4 (2cm/L H2O2), T5 (4cm/L H2O2), T6 (T2+T4), T7 (T2 + T5), T8 (T3+ T4), T9 (T3+ T5).

Spraying combinations of both substances was more effective in increasing nitrogen, potassium, iron, and manganese leaf contents. The combination of the higher rate of both substances (T9) has a superior effect, as it has recorded the highest values of canopy volume, and increased the accumulation of N, K, and Fe in leaves. While the lowest values were recorded from untreated trees during experimental seasons. Data in Table (2) showed that foliar spraying with the studied substances increased the leaf nitrogen content. T9 has the highest N value (2.120 & 2.130%), followed by T8 (2.087%) in the first season and T6 (2.107%) in the second one. On the contrary, the lowest leaf nitrogen content (1.890 & 1.990%) was recorded in untreated trees.

Trees subjected to T9 have the highest K content (1.230 & 1.250%), followed by T4 (1.200%) in the first season and T7 (1.227%) in the second one, while, the control treatment had the lowest significant values (1.120 & 1.163%).

The data in Table (2) revealed that foliar application of T7 significantly increased P concentration in acid lime leaves and recorded the highest values (0.133 &0.136%), followed by T8 (0.131 & 0.134%), whereas T2 recorded the lowest values (0.122 &0.125%). Other treatments had intermediate values of studied nutrients in both seasons.

Variance treatments caused significant differences in leaf Zn content compared to controls. Whereas trees subjected to T6 recorded the highest leaf content of Zn (34.54 & 35.72 mg/kg), followed by T7 (33.77 & 34.12 mg/kg). Untreated trees recorded the lowest values (30.34 & 30.61 mg/kg) during both seasons.

Regarding leaf Mn content, the combination of a high rate of CHI and a low rate of H_2_O_2_ (T8) recorded the highest values (39.70 & 38.33 mg/kg) compared to other treatments in both seasons, while, untreated trees (T1) had the lowest values (31.92 & 31.29 mg/kg).

Data presented in Table (3) indicated that all treatments statistically increased tree yield (kg/tree) compared with the control treatment during the experiment. The maximum tree yield (23.56 and 29.64 kg/tree) was produced from trees that received 100 ppm CHI and 2 cm/L H_2_O_2_ (T6), the control treatment (T1) had the lowest values (14.03 & 18.36 kg/tree) in this respect.

**Table 3:** Effect of variance treatments on yield parameters and physical fruit quality of lime trees.

Treatments	Yield/Tree (kg)		Total yield (ton/ha)		Fruit weight (g)		Fruit size (mm)		Fruit density		Juice Weight (g)	
	2021	2022	2021	2022	2021	2022	2021	2022	2021	2022	2021	2022
T1	14.03E	18.36 H	6.04 F	6.13 F	26.51 B	26.14 BC	21.17 AB	23.00 A	1.274BC	1.158 A	17.50 CD	17.08 A
T2	17.24D	20.59G	6.17 EF	6.60 EF	26.22 B	26.06 C	23.67 AB	22.60 A	1.132 C	1.161 A	22.60 AB	22.22 A
T3	17.50D	22.04E	6.66 DE	7.01 DE	28.14 AB	29.76 A	23.17 AB	26.37 A	1.228BC	1.132 A	17.30 CD	18.33 A
T4	17.20D	20.89FG	6.87 D	6.69 EF	28.20 AB	29.27 AB	24.83 AB	26.73 A	1.174 C	1.146 A	15.71 D	16.17 A
T5	17.26D	21.10 F	6.96 CD	7.38 CD	28.46 AB	29.96 A	25.67 AB	26.50 A	1.132 C	1.131 A	17.39 CD	18.30 A
T6	23.56 A	29.64 A	8.15 A	9.16 A	30.73 A	31.53 A	26.83 A	28.83 A	1.159 C	1.123 A	24.09 A	24.77 A
T7	21.50B	26.11B	7.50 BC	7.97 BC	28.67 AB	30.64 A	21.50 AB	28.13 A	1.344B	1.099 A	22.01 AB	21.89 A
T8	19.73 C	25.50 C	7.62 AB	8.09 B	29.78 AB	30.13 A	19.83 B	27.50 A	1.523 A	1.108 A	19.38 BCD	23.71 A
T9	19.92 C	24.54D	7.59 AB	8.19 B	29.43 AB	28.97 ABC	26.50 A	26.30 A	1.128 C	1.114 A	20.26 ABC	19.99 A

*Values in the same column followed by the same letter(s) do not significantly differ from each other according to Duncan’s multiple range test at 5% level.

* T1 (Control), T2 (100 ppm CHI), T3 (200 ppm CHI), T4 (2cm/L H2O2), T5 (4cm/L H2O2), T6 (T2+T4), T7 (T2 + T5), T8 (T3+ T4), T9 (T3+ T5).

Yield per hectare has the same trend, whereas T6 recorded the maximum yield (8.15 & 9.16 ton/ha) followed by T7 (7.50 & 7.97 ton/ ha), while the lowest total yield (6.04 & 6.13 ton/ha) was recorded with control treatments. The weight of a single lime fruit in each treatment was significantly increased compared to control in both seasons except for T2. The maximum values of fruit weight (30.73 and 31.53 g), fruit size (26.83 and 28.83 mm), and juice weight (24.09 and 24.77 g) were recorded due to a spray with 100 ppm CHI and 2 cm/L H_2_O_2_ (T6) in both seasons. Conversely, trees subjected to T2 produced the smallest fruit weight (26.22 & 26.06 g) and fruit size (23.67 & 22.60 mm), however, the control treatment recorded the lowest value of juice weight (17.50 & 17.08 g) during the experimental seasons, respectively. These results indicate that foliar application of CHI with H_2_O_2_ could have positive effects on the physical quality of lime fruit.

**Fig. 2:**
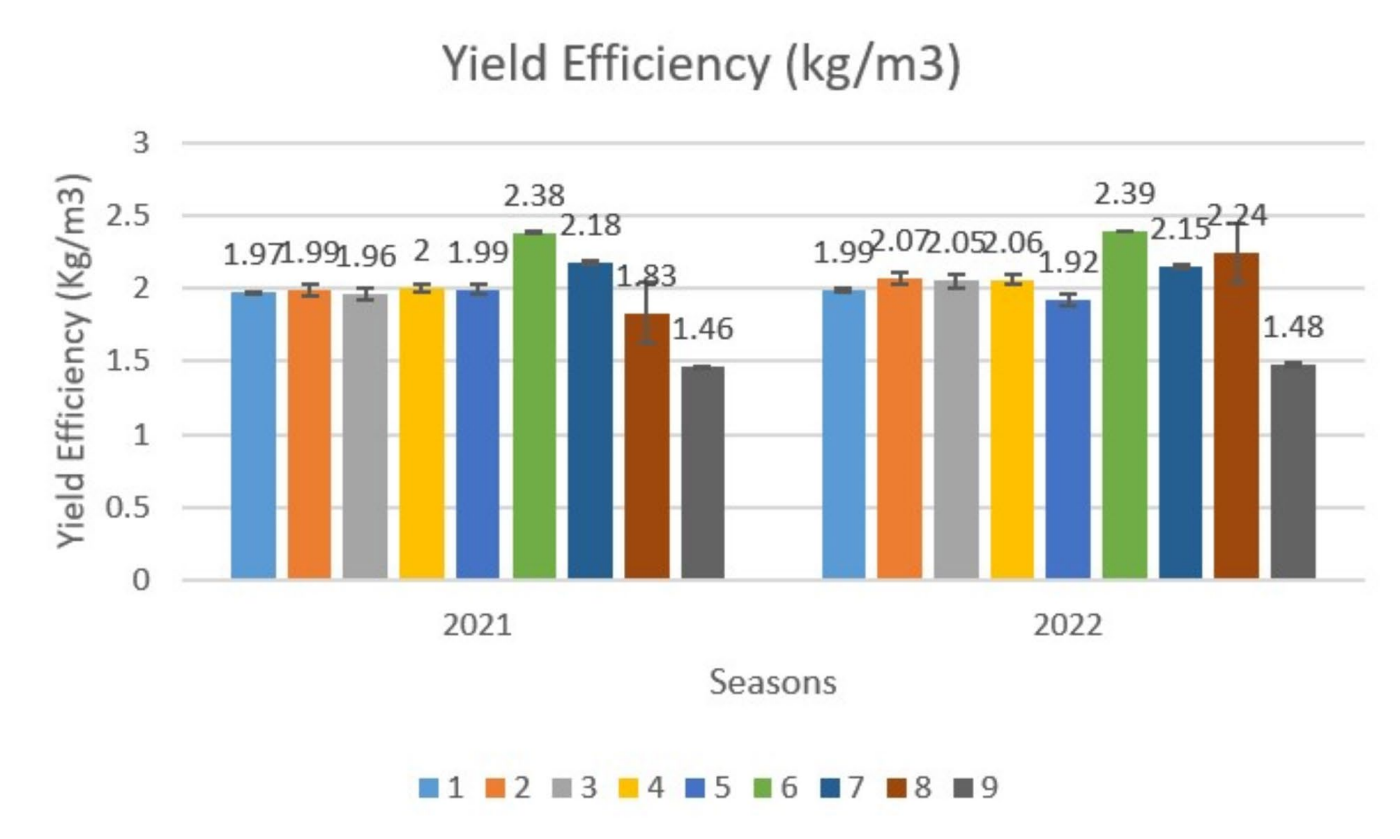
Effect of variance treatments on yield efficiency of lime trees. * T1 (Control), T2 (100 ppm CHI), T3 (200 ppm CHI), T4 (2cm/L H2O2), T5 (4cm/L H2O2), T6 (T2+T4), T7 (T2 + T5), T8 (T3+ T4), T9 (T3+ T5).

Concerning yield efficiency (kg/m^3^) Fig. (2) shows that, there is a variation between different treatments, whereas T6 recorded the highest values (2.38 & 2.39) followed by T7 (2.18) in the first season and T8 (2.24) in the second one, while T9 recorded the lowest values (1.46 &1.48) during the experimental seasons. Furthermore, no significant difference was detectable between T1, T2, T3, T4, and T5 in the first season and between T1, T2, T3, and T4 in the second one.

**Fig. 3:**
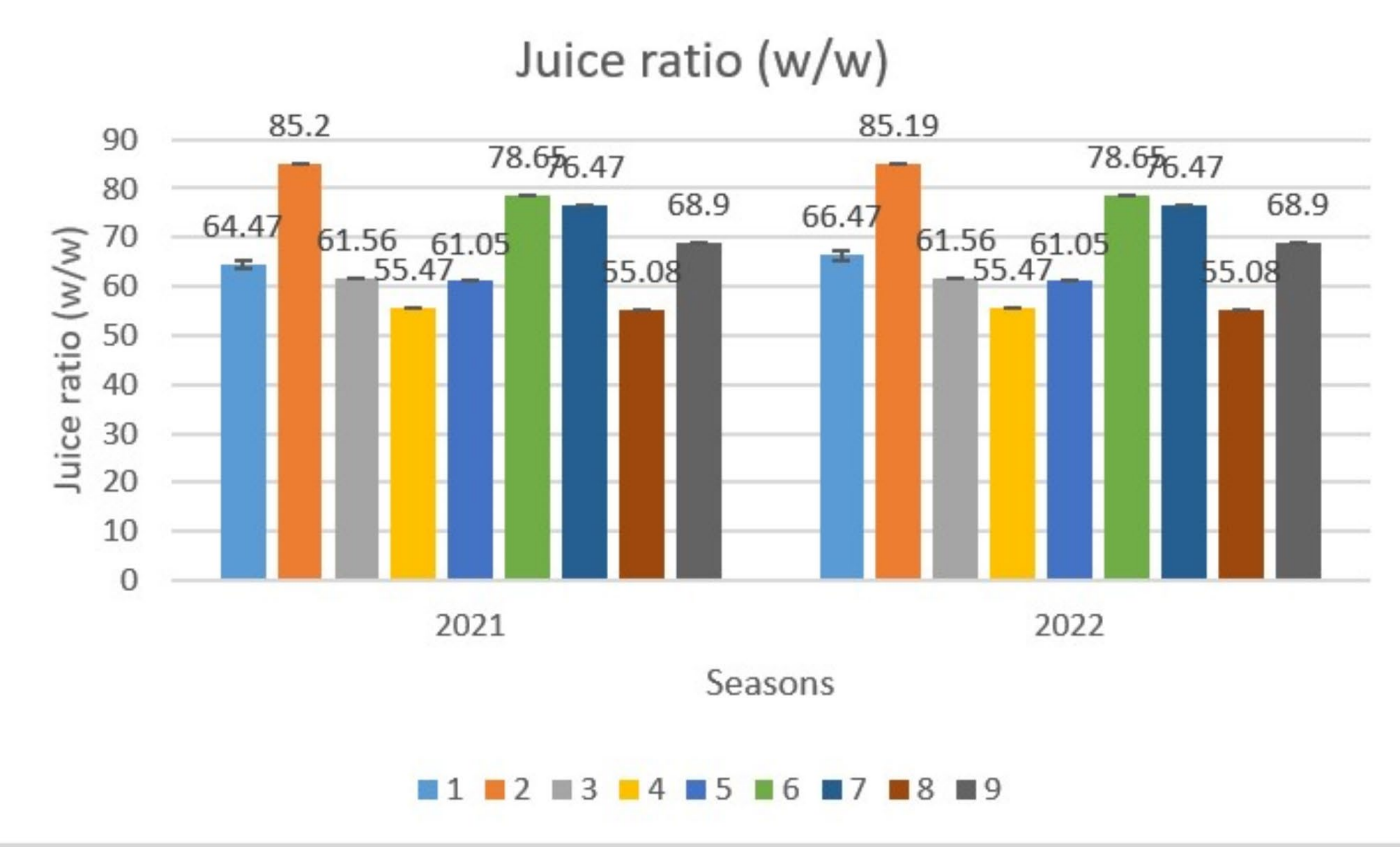
Effect of variance treatments on juice ratio (w/w) of lime fruit. * T1 (Control), T2 (100 ppm CHI), T3 (200 ppm CHI), T4 (2 cm/L H_2_O_2_), T5 (4 cm/L H_2_O_2_), T6 (T2 + T4), T7 (T2 + T5), T8 (T3 + T4), T9 (T3 + T5).

Regarding effect of CHI and H_2_O_2_ treatments on juice ratio (W/W) of the acid lime fruits, Fig. (3) showed that the positive effect of different treatments, especially with the low concentrate of CHI (T2) compared to other treatments. At the same time, T3 recorded the lowest value (61.56%) in the first season and T4 (55.47%) in the second one.

Concerning internal fruit characters, the data in hand (Table 4) cleared an uptrend compared with the control trees excluding T2. On the contrary, the total acidity had the opposite trend, and the control treatment recorded the highest value. However, there are positive effects of CHI and H_2_O_2_ on the internal quality of acid lime fruits, and this can help improve lime’s fruit-added value.

For the effects of CHI & H_2_O_2_ applications on the total soluble solid content of lime fruit, data in Table (4) shows that the total soluble solid content increased obviously when compared to the control except for T9, which recorded the lowest values (4.42 & 4.41). However, the highest significant increase was present in T6 (4.92) in the first season and T3 (4.88) in the second one during the experimental seasons, approximately. On the contrary, foliar application of CHI & H_2_O_2_ reduces the total acid content of lime fruits. It was obvious that the T7 caused a significant reduction in the total acidity (6.80 &6.57%) compared to the control or other combinations. While the control treatment recorded the highest values in both seasons (7.68 & 7.63%).

TSS/acidity ratio was affected by variance treatments and increased when compared with the control treatment, which recorded the lowest values (0.597& 0.615%) while T4 had the maximum significant values (0.664%) in the first season and T7 (0.690%) in the second one compared to the control.

Data in Table (4) indicated that vitamin C content of lime fruit was influenced by variance treatments. Trees subjected to T6 have recorded the highest values (36.00 & 36.07 mg/100 g of fruit juice), followed by T9 (35.37 & 34.53 mg/100 g of fruit juice). Untreated trees had the lowest values (29.59 & 28.53 mg/100 g of fruit juice). While there is no significant difference between T2, T3, T4, and T5 treatments in both seasons.

**Table 4:** Effect of variance treatments on internal fruit characters of lime trees.

Treatment	TSS (Brix)		Acidity (% ) Citric acid			TSS/acid %		Vitamin C (mg/100 ml)	
	2021	2022	2021		2022	2021	2022	2021	2022
T1	4.58 B-D	4.72 AB	7.68 A		7.63 A	0.597 C	0.615 D	29.59D	28.53E
T2	4.60 B-D	4.78 AB	7.55 AB		7.45 B	0.609 BC	0.644 CD	29.63D	29.37D
T3	4.50 C-E	4.88 A	7.38 B		7.50 B	0.610 BC	0.654 BC	29.89D	29.57D
T4	4.67 B	4.85 A	7.03 C		7.18 C	0.669 A	0.675 AB	29.67D	29.36D
T5	4.62 BC	4.53 CD	7.53 AB		7.17 C	0.614 BC	0.637 CD	29.77D	29.53D
T6	4.92 A	4.67 BC	7.57 AB		6.83 E	0.651 A	0.683 A	36.00 A	36.07 A
T7	4.47 DE	4.53 CD	6.80 D		6.58 F	0.659 A	0.691 A	34.67 C	33.10 C
T8	4.63 BC	4.46 D	7.05 C		6.97 D	0.656 A	0.640 CD	34.20 C	32.70 C
T9	4.42 E	4.41D	6.87 CD		6.57 F	0.644 A	0.673 AB	35.37B	34.53B
LSD at 5% =								0.4687	0.5389

*Values in the same column followed by the same letter(s) do not significantly differ from each other according to Duncan’s multiple range test at 5% level.

* T1 (Control), T2 (100 ppm CHI), T3 (200 ppm CHI), T4 (2 cm/L H_2_O_2_), T5 (4 cm/L H_2_O_2_), T6 (T2 + T4), T7 (T2 + T5), T8 (T3 + T4), T9 (T3 + T5).

## Discussion

This study is intended to focus on the growth, productivity, and fruit quality of acid lime trees. Data in hand showed that foliar spraying with the studied substances particularly combinations of higher concentrations of CHI and H_2_O_2_ caused significant improvement in the tree canopy and increased the leaf mineral content compared to control trees. This could be due to its role in enhancing water availability and facilitating nutrient absorption. The beneficial effect of CHI on tree productivity may be due to its action as a biostimulant that influences metabolic pathways, promotes cell division and elongation, improves photosynthesis, regulates hormonal balances that promote flowering and fruit set, and reduces fruit drop, thus increasing tree productivity^4^. H₂O₂ acts as a signalling molecule that regulates pathways such as cell proliferation and flower differentiation and oxidizes particular cysteine residues in proteins^24^. Low levels of H₂O₂ are indispensable for signalling and regulating various biological processes. It has a significant role in cell cycle regulation and immune responses, thus enhancing flowering and total yield^7^. The above results supported the proposed hypothesis, whereas treatments of CHI and H₂O₂ have positive effects on tree growth, seasonal yield, and improvement of fruit quality of acid lime. Affirmative responses of yield parameters were recorded due to the application of CHI and H_2_O_2_, whereas, all treatments were effective in enhancing the total yield and fruit quality of lime trees compared with untreated trees. The above results are in agreement with founds on wax apple fruits^14^, on grapes^25^, on mango trees^12^, who reported that the application of H_2_O_2_ increases yield, fruit size, and juice volume. In this regard, previous studies on various fruit trees claimed that foliar application of CHI improved tree growth and increased leaf mineral contents [8 &9] on navel orange trees;^11^ on mango trees;^26^ on sour orange plants; and^27^ on Olive Trees. Furthermore, the application of H_2_O_2_ improves the vegetative growth of mango trees^12^. Results in Table (3) indicated the beneficial effects of CHI and H_2_O_2_ treatments on yield, it could be due to improving fruit set and reducing fruit drop, which consequently increases tree yield. Most treatments improved external fruit characters compared to control, while single applications of CHI or H_2_O_2_ had less effect compared to their combination effect, which significantly improved fruit quality. Results in hand are in agreement with founded on wax apple fruits by^14^, on grapes by^25^, and on mango trees^12^, who reported that the application of H_2_O_2_ increases yield, fruit size, and juice volume. In this study, three indexes (including single fruit weight, fruit volume, and fruit density), are considered the most common and direct parameters used for evaluating the external quality of lime fruit influenced by CHI & H_2_O_2_ treatments. The results showed that most of the indexes in the different treatments increased compared with their corresponding controls, except for fruit weight and fruit volume, whereas the low rate of CHI application (T2) recorded the lowest values. Previous studies dealt with the effects of CHI and H_2_O_2_ on tree productivity and fruit quality. The present results completely correspond to those published on navel orange by [8 &9], on mango by^10^, and on peach by^11^, which indicated that CHI treatments have a significant effect on yield and yield efficiency. Concerning the effect of different substance treatments on fruit quality parameters, data showed positive effects on various external fruit quality parameters. These results are in line with numerous reports on fruit crops, which mentioned that foliar application of CHI enhances tree yield and increases fruit weight and size, i.e. on mango trees by^12^, on peach trees by^11^, and on Navel oranges by^8^. Data in Table (4) showed a positive response of foliar application of CHI and H_2_O_2_ treatment on TSS, acidity, TSS/acid ratio, and Vitamin C content of Acid lime fruit. Treatment of CHI at 100 ppm with 2 ml/L H_2_O_2_ gave the highest values of TSS, TSS/acid ratio, and vitamin C content. The lowest acidity values were recorded by CHI at 200 ppm with H_2_O_2_ at 4 ml/L. Positive effects of exogenous application of substances could be due to accelerated sugars moving from the leaves to fruits during growth stages. These results are in agreement with previous reports on wax apple by^14^, on mango by^10^, and on passion fruit by^28^, who reported that H_2_O_2_ treatments improves fruit quality. Regarding the effect of variance treatments on juice acidity ratio, the obtained results were in line with previous reports on navel orange trees by^8^, on mango trees by^12^, and on peach trees by^11^, whom showed that acidity % was statistically reduced by chitosan foliar application in comparison with control. Furthermore, H_2_O_2_ application decreases the acidity of mango fruits^12^. The present results completely correspond to previous studies that dealt with the effects of CHI and H_2_O_2_ on tree productivity and fruit quality. Who reported that, CHI treatments had positive effects on yield, yield efficiency, and enhanced fruit parameters of numerous fruit crops, such as navel orange by [8 & 9], on mango by [10 &12], and on peach by^11^. Moreover, H_2_O_2_ treatment improves fruit quality and increases TSS, as reported on grapes by^25^, on mango trees by^12^, and on wax apple by^14^.

## Conclusion

Using chitosan and hydrogen peroxide improves the growth and productivity of lemon trees. Therefore, using a foliar combination of the two substances improves the leaf mineral contents, increases the seasonal crop, and enhances fruit quality due to its role in enhancing water retention, enabling nutrient absorption, and improving total soluble solids content. The results showed that the treatment of CHI at 200 ppm + H_2_O_2_ at 4 cm/L (T9) is the most effective in increasing tree canopy and enhancing leaf mineral contents. Treatment with low concentrations of both substances (T6) achieves the highest tree productivity and total yield. The study concluded that foliar application of chitosan and hydrogen peroxide improves tree productivity and fruit characteristics. More investigations are required to evaluate the impact of the recommended treatment with other combinations of chitosan and hydrogen peroxide on the productivity and quality of seasonal yield of acid lime trees in different regions with different cultivation conditions.

## Data Availability

The datasets used and/or analyzed during the current study available from the corresponding author on reasonable request.

## Abbreviations

CHI: Chitosan
H_2_O_2_: Hydrogen peroxide
